# A Rare Case of Uterine Arteriovenous Malformation Following a Cesarean Scar Ectopic Pregnancy

**DOI:** 10.7759/cureus.81497

**Published:** 2025-03-31

**Authors:** Emily J Ditchfield, Suzanne Hill, Lua Saylany, Ajith Samaratunga

**Affiliations:** 1 Obstetrics and Gynaecology, Dubbo Hospital, Dubbo, AUS; 2 Faculty of Medicine and Health, University of Sydney, Sydney, AUS

**Keywords:** acute vaginal hemorrhage, fertility, hysterectomy, rural and remote medicine, uae, uavm, uterine arteriovenous malformation, uterine artery embolization

## Abstract

Uterine arteriovenous malformations (UAVMs) are rare, life-threatening conditions characterized by abnormal communications between uterine arteries and veins, often resulting in severe hemorrhage. This report discusses the case of a 32-year-old woman living in rural Australia, who experienced an acute life-threatening per vaginal bleed following uterine artery embolization (UAE) for an UAVM associated with a cesarean scar ectopic pregnancy. The patient’s rural residence posed significant challenges, including delayed access to specialized care and prolonged travel distances. Despite UAE, she experienced a recurrence of hemorrhage and life-threatening blood loss and required emergency interventions, leading to a hysterectomy. This case highlights the lethality of UAVM, the risks of recurrence after UAE, and the importance of close post-procedural monitoring, particularly for patients in remote areas. It also emphasizes the need to incorporate social factors such as rurality into management decisions, advocating for systemic solutions to support high-risk patients.

## Introduction

Uterine arteriovenous malformations (UAVMs) are a rare but life-threatening cause of hemorrhage in women [1], defined as anomalous communications between arteries and veins, which occur without any mediating capillary network [2]. They can either be congenital or acquired [2]. An acquired UAVM may develop in the healing process following damage to the uterine blood vessels, such as in a cesarean section or dilation and curettage [3], and is more commonly identified in women of reproductive age [1]. They are the result of reactive angiogenesis and typically incorporate intramural arterial branches with the myometrial venous plexus [2]. Women may present with abnormal uterine bleeding (either intermittent or continuous, associated or not associated with menses or a procedure), acute abdominal pain with hemoperitoneum, or with symptoms suggestive of anemia or hypovolemic shock [1]. UAVM may also be detected incidentally during radiological imaging performed for unrelated reasons [1]. Ultrasound is usually the first line for diagnosis, with color Doppler demonstrating high-velocity mixed arterial and venous flow [2]. Gold-standard diagnosis of an arteriovenous malformation (AVM) is CT angiography [2]. Treatment is largely dependent on the hemodynamic stability of the patient, with consideration given to the desire for future fertility, the location and size of UAVM, and the patient’s age [4]. In a hemodynamically unstable patient, control of acute hemorrhage can be attempted with uterine packing, insertion of a Foley catheter, or various other medical treatments [2]. Longer-term management for these patients historically involved a hysterectomy [4]. However, in recent years, uterine artery embolization (UAE) has become more widely available and acceptable [2], and is preferred if seeking to preserve fertility [2]. It is generally regarded as a safe and fast option, with minimal side effects and a quick recovery [2]. However, a recent systematic review by Labarta et al. suggests that, owing to the rarity of UAVM, high-level evidence to guide management is lacking [2], reflected by a wide range of techniques and agents (both permanent and temporary) used for embolization [2]. A primary UAE success rate of 79.2% has been reported [2], but given that the possible outcome of failed embolization is profuse, even catastrophic vaginal bleeding [1], how should we navigate post-embolization monitoring to ensure resolution? The lack of high-level evidence also extends to follow-up protocols, resulting in no standardized approach [2]. However, the risk of recurrence underscores the necessity for vigilant monitoring and ensuring that the patient remains within close proximity to the treating hospital.

Here, we discuss the case of a 32-year old woman, living remotely, who suffered a life-threatening vaginal hemorrhage following recent UAE.

## Case presentation

A 32-year-old female, G4, P1, travelled 300 km, from her rural home to her nearest level five hospital with acute vaginal bleeding three weeks after revision of a cesarean section scar as extended management of a cesarean scar ectopic six months prior. Excluding her obstetric and gynecological history and the presence of anti-M antibodies, the patient had no significant medical history, with no regular medications and no other surgeries apart from those detailed below.

**Figure 1:**
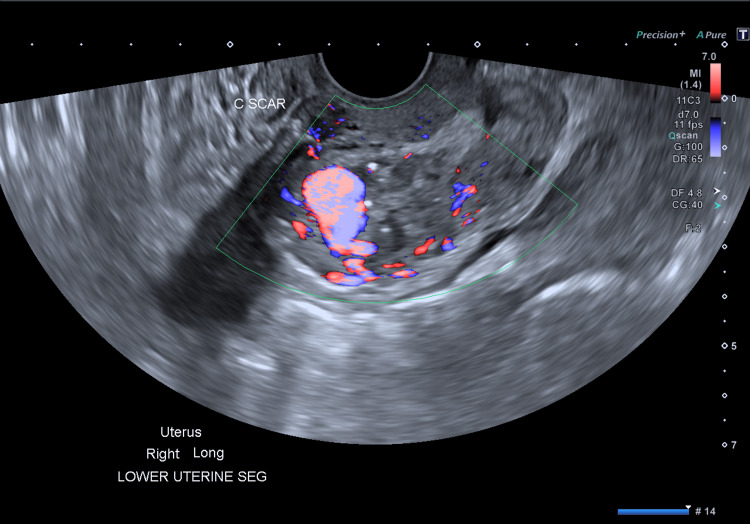
Transvaginal ultrasound of the pelvis demonstrating a 39 × 35 × 29 mm heterogenous mass within the myometrium of the lower uterine segment, arising from the posterior wall. It contains both arterial and venous components with a strong, high-velocity flow.

Three and a half years before her presentation, she underwent a routine lower-segment cesarean section at term, for maternal request, with minimal blood loss and an uncomplicated postoperative recovery. She subsequently experienced a first and second-trimester miscarriage, both requiring dilatation and curettage, and a cesarean scar ectopic, which was managed with a hysteroscopy and removal of products of conception using MyoSure® (Hologic, CA, USA). Following the hysteroscopy, she reported heavy vaginal bleeding, which was initially managed with tranexamic acid. Six months later, she underwent a laparoscopic revision of the cesarean section scar for possible retained products of conception in the context of ongoing vaginal bleeding and a negative pregnancy test. During this procedure, she had an estimated blood loss of approximately 1 L, necessitating conversion to laparotomy to ligate bleeding vessels near the previous scar. Three weeks post surgery, she was again admitted to hospital with heavy vaginal bleeding and was diagnosed with a posterior lower-segment UAVM (39 × 35 × 29 mm) on pelvic ultrasound. Transvaginal ultrasound demonstrated a large caliber, abnormal vessel, containing both arterial and venous components with strong, high-velocity flow [5] (Figure 1).

**Figure 2:**
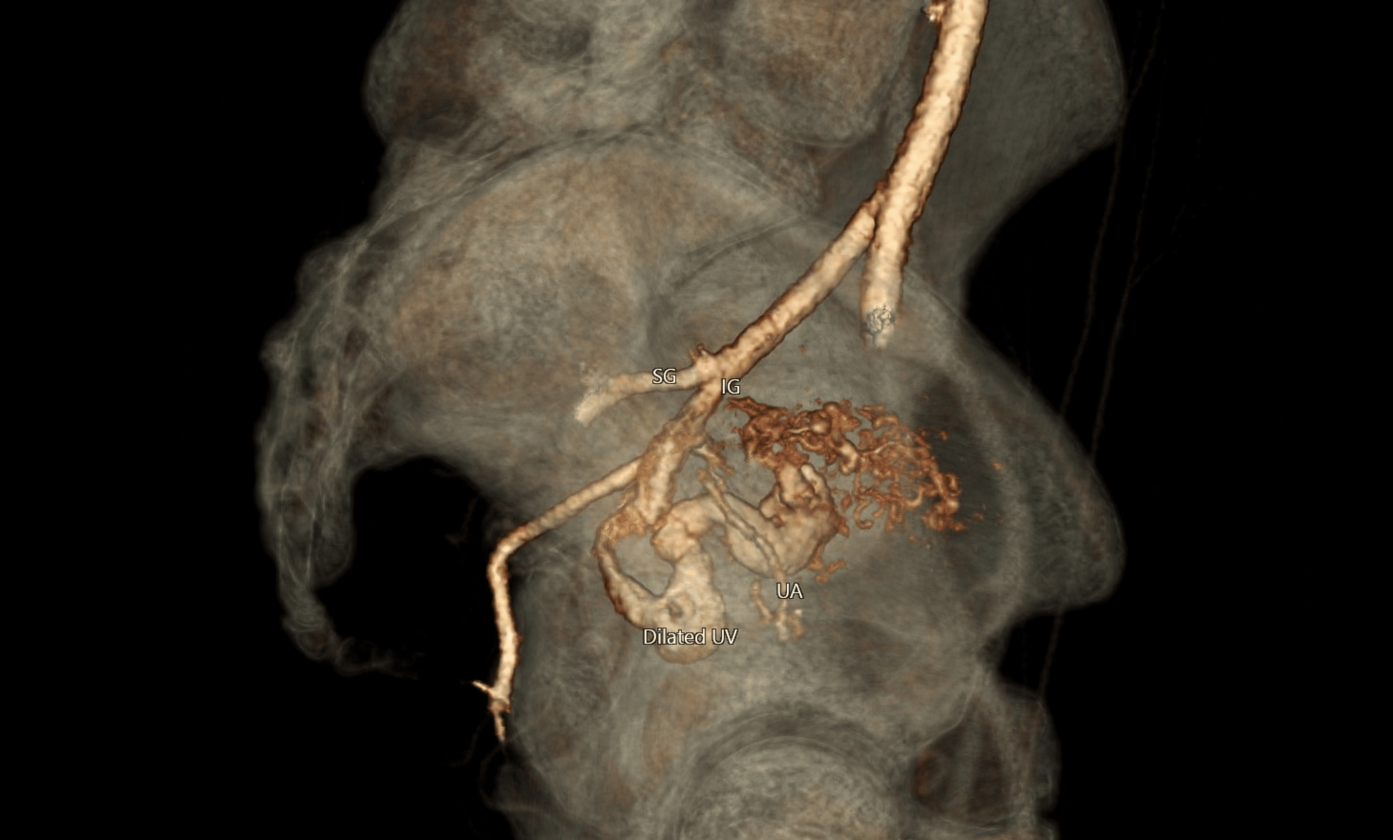
Three-dimensional reconstruction of CT angiogram demonstrating the grossly dilated uterine vein as it flows from the arteriovenous malformation. SG = superior gluteal artery; IG = inferior gluteal artery; UA = uterine artery; UV = uterine vein

During admission, she became hemodynamically unstable secondary to an acute 2 L blood loss and was transferred to a metropolitan level six hospital. The hospital first confirmed the diagnosis of UAVM using CT angiography (Figure 2) before proceeding with UAE.

**Figure 3:**
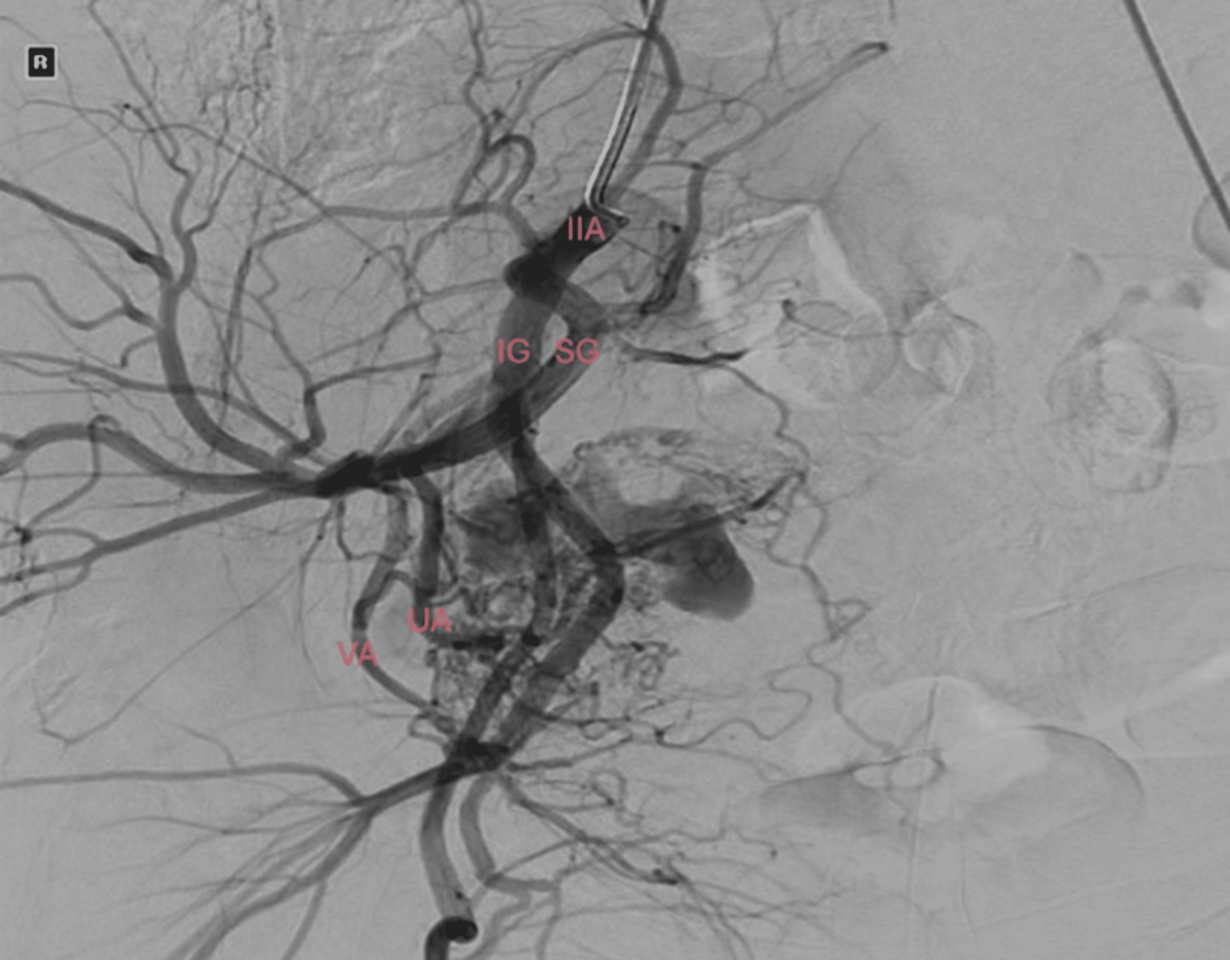
Pre-embolization digital subtraction angiography. IIA = right internal iliac artery; SG = right superior gluteal artery; IG = right internal gluteal artery; UA = right uterine artery; VA = right vaginal artery

**Figure 4:**
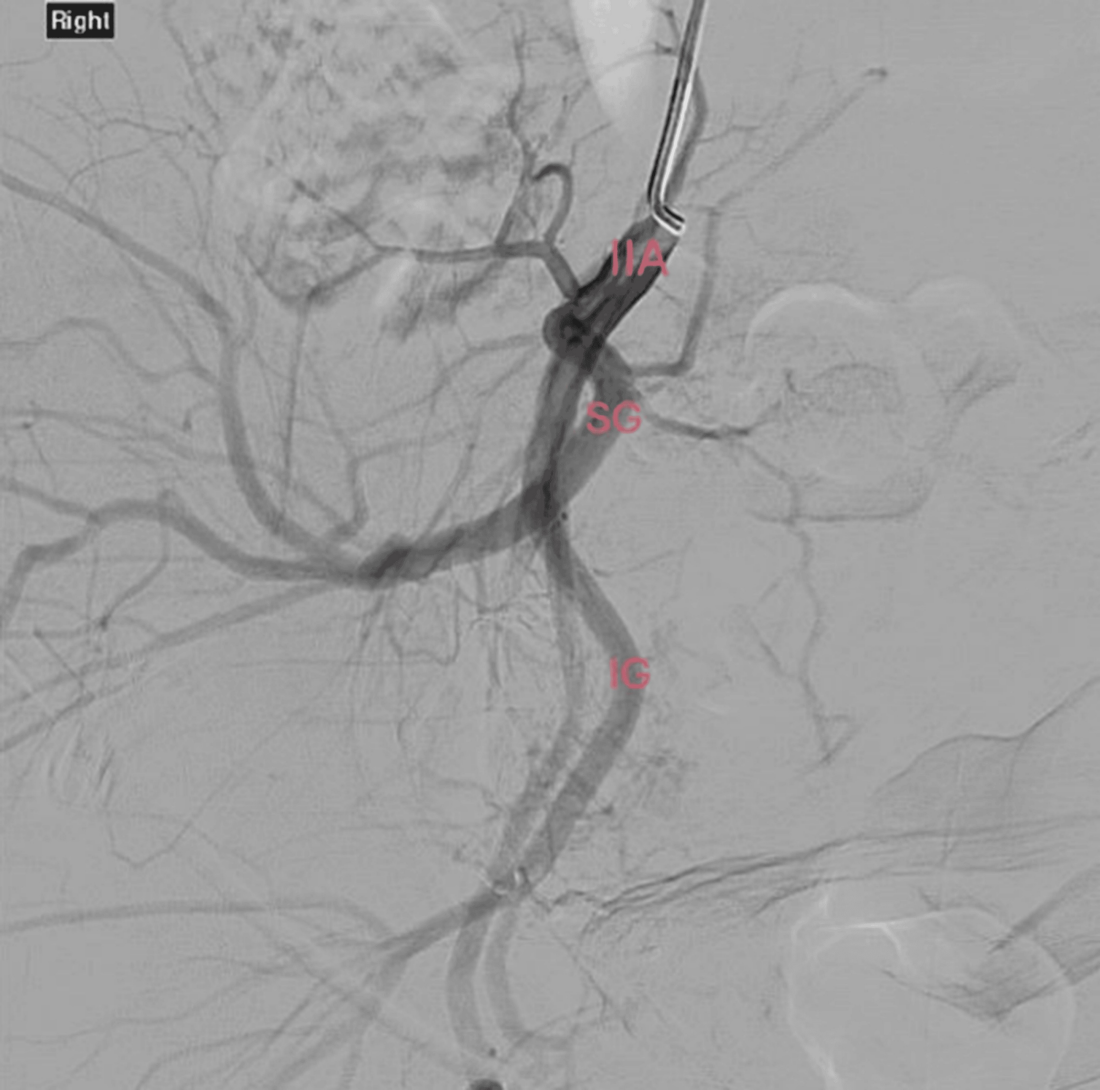
Post-embolization digital subtraction angiography. IIA = right internal iliac artery; SG = right superior gluteal artery; IG = right internal gluteal artery

The embolization was performed under digital subtraction angiography (DSA) through the left common femoral artery using thick Gelfoam® (Pfizer, MI, USA) slurry, a temporary embolic agent. Feeders to the UAVM had been identified from the right uterine artery and the right vaginal artery, both of which were selectively treated (Figures 3, 4). Possible gonadal supply was also noted; however, this was not treated due to concern about fertility preservation.

The female was placed on four hours of bed rest post-procedure and discharged the next day, following a pelvic ultrasound, which showed no features of AVM. Progress imaging in six weeks was recommended to monitor the resolution of the AVM.

She travelled 650 km on her return home on the day of discharge, where she remained for nine days until she presented to her nearest level five hospital with another episode of acute vaginal bleeding. On initial review in the emergency department, she was hemodynamically stable, with speculum examination revealing old blood. Following discussion with the hospital that managed her UAE, a return transfer was agreed upon, and she was commenced on 1 g of tranexamic acid QID. While awaiting the transfer, approximately four hours later, she experienced brisk vaginal bleeding. The on-call registrar quickly applied manual pressure through the vagina with temporary cessation of bleeding. She was then transferred to the theater for an emergency examination under anesthesia, where a Foley catheter was inserted intrauterine and filled with 15 mL of saline, with complete resolution of bleeding. However, by this time, she had an estimated blood loss of approximately 2 L and was hemodynamically unstable. She required three units of packed red blood cells, two units of fresh frozen plasma, and remained intubated during air medical retrieval, which facilitated immediate interhospital transfer. The presence of anti-M antibodies in her blood complicated resuscitative efforts with limited supply of M antigen-negative red blood cells available at the level five hospital.

Following transfer to the level six hospital, she was extubated within 24 hours but remained in the intensive care unit. Extensive counseling was conducted with the patient, partner, and multidisciplinary team of interventional radiologists and gynecologists regarding options for management. Namely, two options were proposed: further trial of UAE or hysterectomy. Ultimately, the patient’s decision was for a hysterectomy. This request was partly owing to the patient’s trauma associated with two episodes of critical hemorrhage requiring urgent surgical management and interhospital transfer. Had access to effective management for a possible recurrence been more readily available, further attempts at UAE and preservation of her fertility might have presented a more viable option.

## Discussion

We have described the case of a 32-year-old woman, living in rural Australia, who experienced a life-threatening hemorrhage following failed UAE for an UAVM.

Overall, 28% of Australia’s population lives in rural and remote areas, facing many challenges, including limited access to health care, options for education, and difficulty in securing employment [6]. Higher rates of potentially avoidable deaths and deaths occurring at a younger age are more apparent in these areas, with an overall mortality rate in very remote areas almost one and a half times higher than metropolitan populations [6]. UAVMs are reported as a life-threatening condition, with the majority of patients included in Labarta et al.’s systematic review reporting “persistent and uncontrollable profuse vaginal bleeding” as their main symptom [2]. An UAVM poses such a significant threat because blood is shunted from the high pressure of the uterine artery to the low pressure of the myometrial veins, rupturing the weak walls of these vessels. In remote areas, hundreds of kilometers away from a hospital with facilities adequate to prevent exsanguination, this could rapidly lead to a high burden of morbidity and mortality.

Regarding treatment, although some exceptions exist related to the size and location of UAVM, hysterectomy is generally accepted as curative [1]; however, it results in infertility, which can have its own associated mental health sequelae [7]. Attempts at medical management for the preservation of fertility are generally reserved for patients with asymptomatic UAVM or those who present with mild hemorrhage. These options include methylergonovine maleate, gonadotropin-releasing hormone analogs, and Danazol [8]. However, they unfortunately have a high rate of failure and persistent bleeding [8]. For patients who have experienced more severe hemorrhage, UAE remains the treatment of choice in women of reproductive age [1]. However, pregnancy following UAE is not without risk, with studies reporting complications, including spontaneous abortion [2], placenta accreta spectrum, and postpartum hemorrhage [8]. A success rate of only 79.2% [2] also suggests a 20.8% risk of possible recurrence of vaginal hemorrhage. The lack of evidence to guide monitoring for resolution following UAE [2] and when or how recurrence may develop makes it difficult to establish safety on discharge.

Recently, some centers have reported success using hysteroscopy to resect AVMs while others have attempted the same laparoscopically [8]. For large AVMs or those with significant bleeding potential, the surgeons would need to possess superior confidence in identifying the relevant anatomy and managing potentially copious bleeding for this to be successful and safe.

## Conclusions

UAVMs are rare but potentially life-threatening conditions that require timely diagnosis and tailored management. This case illustrates the complexities of treating UAVM, particularly in patients residing in rural and remote areas where limited access to advanced healthcare treatment options poses significant challenges. While fertility preservation is a critical consideration, it should not overshadow the primary objective of safeguarding the patient’s life and health. The risk of recurrence following UAE highlights the need for close post-procedural monitoring and the establishment of robust follow-up protocols, especially for patients in remote settings. Strategies such as providing temporary accommodation near tertiary care facilities, shared medical records systems, and upskilling community health workers on the management of specific complications could mitigate some of the risks associated with rurality. Furthermore, increased efforts are needed to enhance the availability of high-level evidence to guide UAVM management and post-treatment care while acknowledging the limited data available. Ultimately, addressing the interplay between medical, social, and geographical factors is essential to improving outcomes and equity in care for patients with UAVM.
